# Aβ misfolding in blood plasma is inversely associated with body mass index even in middle adulthood

**DOI:** 10.1186/s13195-021-00889-2

**Published:** 2021-08-30

**Authors:** Tobias Möllers, Hannah Stocker, Laura Perna, Andreas Nabers, Dan Rujescu, Annette M. Hartmann, Bernd Holleczek, Ben Schöttker, Klaus Gerwert, Hermann Brenner

**Affiliations:** 1 Division of Clinical Epidemiology and Aging Research, German Cancer Research Center, Im Neuenheimer Feld 581, Heidelberg, Germany; 2 Network Aging Research, Heidelberg University, Bergheimer Straße 20, Heidelberg, Germany; 3 Medical Faculty, Heidelberg University, Im Neuenheimer Feld 572, Heidelberg, Germany; 4 Department of Translational Research in Psychiatry, Max Planck Institute of Psychiatry, Kraepelinstraße 2-10, München, Germany; 5 Department of Biophysics, Ruhr-University Bochum, Universitätsstraße 150, Bochum, Germany; 6 Department of Biophysics, Center for Protein Diagnostics (ProDi), Biospectroscopy, Ruhr-University Bochum, Universitätsstraße 150, Bochum, Germany; 7 University Clinic and Outpatient Clinic for Psychiatry, Psychotherapy and Psychosomatics, Martin-Luther-University Halle-Wittenberg, Julius-Kühn-Straße 7, Halle (Saale), Germany; 8 Saarland Cancer Registry, Präsident-Baltz-Straße 5, Saarbrücken, Germany

**Keywords:** Alzheimer’s disease, Amyloid beta misfolding, BMI, cohort study

## Abstract

**Background:**

To understand the potential for early intervention and prevention measures in Alzheimer’s disease, the association between risk factors and early pathological change needs to be assessed. Hence, the aim of this study was to determine whether risk factors of Alzheimer’s clinical syndrome (clinical AD), such as body mass index (BMI), are associated with Aβ misfolding in blood, a strong risk marker for AD among older adults.

**Methods:**

Information on risk factors and blood samples were collected at baseline in the ESTHER study, a population-based cohort study of older adults (age 50–75 years) in Germany. Aβ misfolding in blood plasma was analyzed using an immuno-infrared-sensor in a total of 872 participants in a nested case-control design among incident dementia cases and matched controls. Associations between risk factors and Aβ misfolding were assessed by multiple logistic regression. For comparison, the association between the risk factors and AD incidence during 17 years of follow-up was investigated in parallel among 5987 cohort participants.

**Results:**

An inverse association with Aβ misfolding was seen for BMI at age 50 based on reported weight history (aOR 0.64, 95% CI 0.43–0.96, *p* = 0.03). Similar but not statistically significant associations were seen for BMI at baseline (i.e., mean age 68) and at age 40. No statistically significant associations with Aβ misfolding were found for other risk factors, such as diabetes, smoking, and physical activity. On the other hand, low physical activity was associated with a significantly reduced risk of developing clinical AD compared to physical inactivity.

**Conclusions:**

Our results support that AD pathology may be detectable and associated with reduced weight even in middle adulthood, many years before clinical diagnosis of AD. Physical activity might reduce the risk of onset of AD symptoms.

**Supplementary Information:**

The online version contains supplementary material available at 10.1186/s13195-021-00889-2.

## Background

The development of Alzheimer’s disease (AD) is characterized by early pathological change, specifically amyloid beta (Aβ) deposits and tau tangles in the brain, followed by dementia symptoms, which may occur 15 to 20 years after the initial onset of the disease [1]. Considering the biological construct of AD, it is particularly important to assess the associations of modifiable risk factors with markers of early pathological change.

One aspect of pathological change in AD includes the structural changes of the Aβ peptide, in which its folds are altered from healthy monomeric predominantly disordered or partly α-helical to pathological β-sheet-enriched secondary structures [2]. This is also known as misfolding. Once β-sheet-enriched structures aggregate, they can form soluble toxic oligomers and macroscopically visible amyloid plaques, which are thought to contribute to AD neurodegeneration [3, 4]. The process of misfolding causes a shift in the overall secondary structure distribution within the total Aβ fraction in cerebrospinal fluid (CSF) and blood plasma. Structural misfolding of Aβ in blood plasma can be measured by an immuno-infrared-sensor (iRS) [5, 6]. In previous studies, we have shown that Aβ misfolding in blood plasma is correlated to CSF AD biomarkers and amyloid PET imaging and is highly predictive of Alzheimer’s clinical syndrome (clinical AD) many years before clinical diagnosis [6–8]. Most recently, we were able to show that Aβ misfolding in blood plasma might be an early risk marker of AD that is independent of age [9].

The relationship between common risk factors of clinical AD, such as physical activity or body mass index (BMI), and Aβ misfolding in blood plasma remains unknown to date. A particularly interesting risk factor is obesity. There have been discussions about the “obesity-paradox” where late-life obesity is associated with a lower risk of developing clinical AD as well as studies reporting an association of higher late-life BMI with lower Aβ burden [10, 11].

Therefore, the aim of this study was to assess the association of BMI and other common clinical AD risk factors with Aβ misfolding within a community-based cohort study of older adults. The associations between these risk factors and the incidence of clinical AD were investigated in parallel for comparison.

## Materials and methods

### Study design and population

The study population consists of participants of the ongoing population-based prospective ESTHER cohort study (Epidemiologische Studie zu Chancen der Verhütung Früherkennung und optimierten Therapie chronischer Erkrankungen in der älteren Bevölkerung) [6, 12, 13]. People aged 50–75 years attending a general health examination were recruited by their general practitioners (GPs) in a statewide study in Saarland, Germany, from 2000 to 2002. Standardized self-administered health questionnaires were filled out by participants, who also provided blood samples, including heparin plasma samples (stored at −80 °C). The GPs provided further medical information and comprehensive follow-ups were conducted through participants and GP questionnaires 2, 5, 8, 11, 14, and 17 years after recruitment. Information on vital status and causes of death was obtained from population registries and local health authorities. The ESTHER study was approved by the Ethics Committee of the Medical Faculty of Heidelberg University and the Physicians’ Board of Saarland.

At baseline, 9940 participants were included in the ESTHER study. In a nested case-control approach, Aβ misfolding was measured in blood plasma among cases with a GP reported diagnosis of dementia up to the 14-year follow-up and controls without dementia (i.e., absence of any dementia diagnosis as specified by the participants’ physicians) diagnosis that were matched to the cases by sex, age, and education as previously described [6]. Our main analysis is based on 872 participants (*n* = 167 dementia cases, *n* = 705 participants without dementia diagnosis) in whom measurements of Aβ misfolding were performed.

**Fig. 1:**
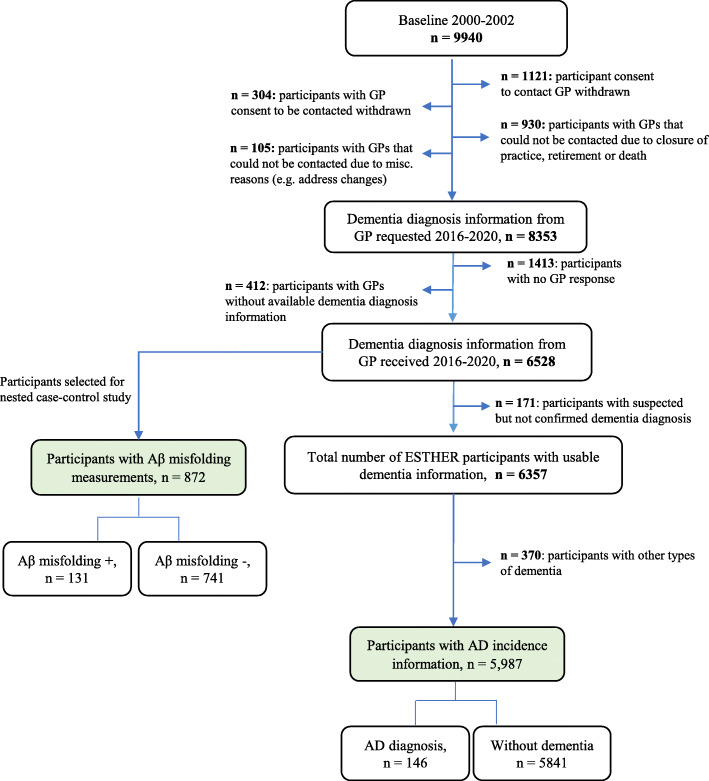
Participants from the ESTHER prospective cohort study included in analyses

For comparison, we conducted a secondary analysis including 5987 participants with available information regarding AD diagnosis or confirmed lack of dementia diagnosis at the 17-year follow-up (Fig. 1). Information on clinical AD diagnosis was provided by the participants’ GPs during the 14- and 17-year follow-ups as previously reported [13]. In short, GPs were asked to fill out questionnaires regarding any dementia diagnosis and type of dementia of the participants and provide all available medical records from specialists such as neurologists or psychiatrists. The National Institute on Aging and the Alzheimer’s Association or the International Working group-2 criteria are recommended for clinical AD diagnosis in Germany [14–16].

### Biomarkers and risk factors

The blood plasma samples used in this study were collected at baseline and Aβ misfolding was assessed as previously reported [6, 7]. Briefly, soluble Aβ peptides were extracted from blood plasma, which was acquired, processed, and frozen at baseline, and alterations in the Aβ peptide secondary structure distribution were measured for each participant with an immuno-infrared-sensor (WO 2015121339 A1), whose details also have been reported elsewhere [5, 6, 17]. In agreement with the previously validated spectral threshold, participants with a cutoff of < 1642 cm^−1^ were considered to have increased Aβ misfolding [6].

*APOE* genotyping was performed using Taqman SNP genotyping assays with genotypes analyzed in an endpoint allelic discrimination read using a PRISM 7000 Sequence detection system (Applied Biosystems, Foster City, CA). Participants with ≥1 *APOE* ε4 allele were considered *APOE* ε4 positive (*APOE* ε4+).

Additional risk factors ascertained at baseline included age, sex, educational level, smoking (never, former, current), physical activity (inactive < 1 h of physical activity/week, low ≥ 1 h of physical activity/week but < 2 h of vigorous and < 2 h of light physical activity/week, medium/high ≥ 2 h of light and ≥ 2 h of vigorous physical activity/week), diabetes (physician diagnosis or use of glucose-lowering drugs), and body mass index (BMI). BMI was based on self-reported measurements of height and weight because this was the more complete assessment at baseline. Height and weight were also measured by the GPs during the health check-up and the measurements had very high agreement with the self-reported information: 95.8% for height within ±5 cm range and 96.5% for weight within ±5 kg range. In addition, BMI calculated from retrospectively reported weights at the age of 50 and 40 years was considered. Based on the limited number of participants in the underweight and obesity groups, BMI was considered dichotomously as < 25.0 (underweight/normal weight) and ≥ 25.0 kg/m^2^ (overweight/obesity) as well as continuously per 5 kg/m^2^.

In addition to single risk factors, a previously established 34-item frailty index (FI) based on the accumulation of deficits approach, with items including comorbidities (e.g., heart failure, stroke), self-rated health, difficulties in activities of daily living, and the presence of symptoms such as insomnia, was utilized [18]. Considered variables had to be associated with health status, accumulate with age, do not saturate too early, have more than 1% prevalence, and should cover a range of health problems and disabilities. This frailty index quantifies frailty as the ratio of present deficits divided by the total number of deficits considered. Cutoff points distinguishing frail and non-frail participants were derived by assessing the 10-year mortality risk with 9 pre-defined cutoff points. Non-significant strata were combined, and the following cutoff points were established: FI 0 to ≤ 0.20 for non-frail, FI 0.21 to < 0.45 for pre-frail, and FI ≥ 0.45 for frail participants, respectively [18].

### Statistical methods

This study included two analyses: (1) the main cross-sectional analysis investigating the association between BMI and other modifiable risk factors of clinical AD with Aβ misfolding measured in blood plasma and (2), for comparison, a longitudinal analysis investigating the association between the abovementioned risk factors and incidence of clinical AD within 17 years of follow-up. The adjusted analysis included all variables mentioned in the risk factors section above.

In the cross-sectional analysis, *APOE* ε4 had the highest percentage of missing values (13.4%). Hence, multiple imputation for data missing at random with thirteen imputations was done using the Markov chain Monte Carlo method utilizing all variables listed in Tables 1 and 2 including Aβ misfolding status [19]. Multiple logistic regression utilizing the imputed datasets, with Aβ misfolding status as the dependent variable, was used to calculate odds ratios (OR) with 95% confidence intervals (CI). The adjusted analysis included all variables mentioned in the risk factors section above.

**Table 1:** Participant characteristics of the nested case-control study within ESTHER and of ESTHER participants with confirmed Alzheimer’s disease status at the 17-year follow-up

		Nested case-control study within ESTHER		ESTHER participants with confirmed AD status from the 17-year follow-up	
Characteristics at baseline		Aβ misfolding+ *n* (%)	Aβ misfolding− *n* (%)	Alzheimer’s disease diagnosis *n* (%)	Participants without dementia diagnosis *n* (%)
Total		131 (15.0)	741 (85.0)	146 (2.4)	5841 (97.6)
Age	Mean ± SD	68.3 ± 4.9	68.5 ± 4.7	66.7 ± 5.1	61.3 ± 6.5^***^
	50–64	27 (20.6)	137 (18.5)	48 (32.9)	3902 (66.8)^***^
	65–69	39 (29.8)	236 (31.9)	45 (30.8)	1259 (21.6)^***^
	70–75	65 (49.6)	368 (49.7)	53 (36.3)	680 (11.6)^***^
Sex	Female	68 (51.9)	435 (58.7)	89 (61.0)	3184 (54.5)
	Male	63 (48.1)	306 (41.3)	57 (39.0)	2657 (45.5)
Education	≤ 9 yrs	115 (87.8)	645 (88.2)	117 (82.4)	4154 (72.7)^*^
	≥ 10 yrs	16 (12.2)	86 (11.8)	25 (17.6)	1561 (27.3)^*^
*APOE* ε4+	No	81 (66.9)	484 (74.7)	64 (50.0)	3926 (75.0)^***^
	Yes	40 (33.1)	164 (25.3)	64 (50.0)	1309 (25.0)^***^
Diabetes	No	100 (76.3)	586 (79.1)	116 (79.5)	4950 (85.7)^*^
	Yes	31 (23.7)	155 (20.9)	30 (20.6)	825 (14.3)^*^
Physical activity	Inactive	35 (26.7)	243 (32.8)	49 (33.6)	1061 (18.2)^***^
	Low	68 (51.9)	352 (47.6)	57 (39.0)	2648 (45.4)^***^
	Medium/high	28 (21.4)	145 (19.6)	40 (27.4)	2118 (36.4)^***^
BMI	Mean ± SD	27.1 ± 3.8	28.0 ± 4.2^**^	27.2 ± 3.9	27.7 ± 4.5
	< 25	37 (29.1)	171 (23.8)	45 (31.5)	1612 (28.1)
	≥25	90 (70.9)	548 (76.2)	98 (68.5)	4127 (71.9)
Smoking	Never	66 (50.8)	396 (55.7)	83 (58.9)	2891 (50.6)
	Former	47 (36.2)	234 (32.9)	42 (29.8)	1933 (33.8)
	Current	17 (11.4)	81 (11.4)	16 (11.4)	892 (15.6)
Frailty Index	Mean ± SD	0.3 ± 0.1	0.3 ± 0.2	0.3 ± 0.2	0.2 ± 0.1^***^
	Non-frail 0 to ≤0.20	31 (34.4)	175 (39.7)	39 (39.4)	2298 (53.3)^**^
	Pre-frail > 0.20 to < 0.45	50 (55.6)	203 (46.0)	45 (45.5)	1697 (39.4)^**^
	Frail ≥0.45	9 (10.0)	63 (14.3)	15 (15.2)	315 (7.3)^**^

^*^p-value of <.05

^**^p-value of <.01

^***^p-value of <.001

*p*-values derived from the *t*-test for continuous and chi-square tests for categorical variables

**Table 2:** Distribution of BMI at different time points and its association with Aβ misfolding and clinical Alzheimer’s disease

		Aβ misfolding				Clinical Alzheimer’s disease			
Time point		*N*_total_ (col %)	*N*_Abeta+_ (row %)	OR (95% CI)	*p*-value^b^	*N*_total_ (col %)	*N*_AD_ (row %)	HR (95% CI)	*p*-value^b^
				Adjusted^a^				Adjusted^c^	
Baseline	< 25 kg/m^2^	208 (24.6)	37 (17.8)	Ref.		1657 (28.2)	45 (2.7)	Ref.	
	≥25 kg/m^2^	638 (75.4)	90 (14.1)	0.82 (0.49–1.36)	.4385	4225 (71.8)	98 (2.3)	0.66 (0.43–1.00)	.0501
	Per 5 kg/m^2^	846 (100)	127 (15.0)	0.73 (0.52–1.03)	.0705	5882 (100)	143 (2.4)	0.88 (0.67–1.16)	.3736
At age 50	< 25 kg/m^2^	335 (42.8)	59 (17.6)	Ref.		2341 (41.3)	62 (2.7)	Ref.	
	≥25 kg/m^2^	448 (57.2)	61 (13.6)	0.88 (0.54–1.41)	.5817	3327 (58.7)	73 (2.2)	0.81 (0.54–1.21)	.3048
	Per 5 kg/m^2^	783 (100)	120 (15.3)	**0.64 (0.43–0.96)**	**.0314**	5668 (100)	135 (2.4)	1.13 (0.83–1.53)	.4455
At age 40	< 25 kg/m^2^	468 (61.9)	74 (15.8)	Ref.		3269 (58.3)	76 (2.3)	Ref.	
	≥25 kg/m^2^	288 (38.1)	38 (13.2)	0.87 (0.53–1.42)	.5760	2340 (41.7)	49 (2.1)	1.08 (0.72–1.62)	.7101
	Per 5 kg/m^2^	756 (100)	112 (14.8)	0.80 (0.54–1.19)	.2704	5609 (100)	125 (2.2)	1.13 (0.81–1.56)	.4753

^a^Adjusted for all variables listed in Table 1 and case/control status

^b^*p*-value derived from multiple logistic regression for adjusted odds ratios

^c^Adjusted for all variables listed in Table 1

In the secondary analysis, *APOE* ε4 had the highest percentage of missing values as well (11.6%). Accordingly, multiple imputation for data missing at random with twelve imputations was done using the Markov chain Monte Carlo method utilizing all variables listed in Tables 1 and 2 including AD status [19]. Cox proportional hazards regression utilizing the imputed datasets was used to calculate hazard ratios (HRs) including 95% CIs with the incidence of clinical AD diagnosis as the dependent variable. The censoring dates for these analyses included the date of AD diagnosis, date of death, date of drop out, or date of the 17-year follow-up (date of response from the GP regarding dementia diagnosis status).

Multicollinearity in multivariable models was examined (and found to be of no concern) by the tolerance, variation inflation factor, eigenvalue, and condition index. Participant characteristics were compared utilizing *t*-tests for continuous and chi-square tests for categorical variables. Statistical difference was defined by *p*-values < 0.05 in two-sided testing. All analyses were conducted using SAS software, version 9.4 (SAS Institute, Cary, NC).

## Results

### Sample characteristics

Details regarding the participant characteristics and a flowchart outlining the sample derivation are presented in Table 1 and Fig. 1.

In the main analysis, 872 participants with available Aβ misfolding measurement were included, of whom 131 (15%) were considered to have increased Aβ misfolding. In terms of age, sex, education, and diabetes, the participants with increased and normal Aβ misfolding were fairly similar. *APOE* ε4 allele presence was more prevalent in participants with increased Aβ misfolding. A lower percentage of participants with increased Aβ misfolding had a BMI ≥ 25 kg/m^2^ at baseline than participants with normal Aβ misfolding.

In the secondary analysis, a total of 5987 participants with available information on clinical AD status were included, of whom 146 (2.4%) had a diagnosis of AD. On average, participants who were diagnosed with clinical AD during follow-up were older than participants who remained dementia-free (66.7 vs. 61.3). Incident AD cases were also more commonly female and less educated than those without AD diagnosis (61% vs. 55% and 82.4% vs. 7.3%, respectively). Physical inactivity (33.6% vs. 18.2%) and frailty (15.2% vs. 46.7%) at baseline were more common among participants with a later diagnosis of AD as well.

### Association of BMI with Aβ misfolding and clinical AD

**Fig. 2:**
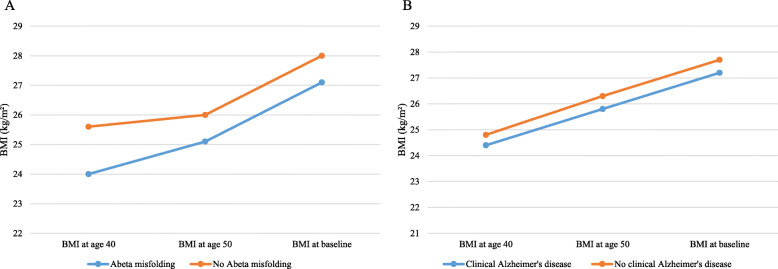
Comparison of mean BMI at different time points between participants with Abeta misfolding and no Abeta misfolding (**A**) and clinical Alzheimer’s disease and no clinical Alzheimer’s disease (**B**), respectively

Figure 2 shows that the mean BMI rose from age 40 to age 50 to baseline among participants included in the cross-sectional and longitudinal analysis. The lower BMI among participants with increased compared to normal Aβ misfolding was much more pronounced than the lower BMI of participants with clinical AD compared to participants without dementia diagnosis. In fact, Table S1 (Suppl. Material) shows that there were no significant differences in mean BMI values for AD cases compared to dementia-free participants at baseline, at the age of 50 or at the age of 40. The mean BMI values were significantly lower for participants with increased Aβ misfolding than for participants with normal Aβ misfolding at each time point (baseline, 27.1 vs. 28.0; age 50, 25.1 vs. 26.0; age 40, 24.0 vs. 25.6 kg/m^2^) (Fig. 2).

Associations of BMI with Aβ misfolding and clinical AD adjusted for all covariates shown in Table 1 are presented in Table 2. Interestingly, there was an association of BMI values with Aβ misfolding at different time points. In detail, an increase of BMI per 5 kg/m^2^ at age 50 was associated with a significantly reduced odds of having increased Aβ misfolding at baseline (OR per 5 kg/m^2^ 0.64, 95% CI 0.43–0.96). Additionally, similar inverse associations were apparent for BMI per 5 kg/m^2^ at baseline and at age 40 and baseline Aβ misfolding (OR_BL_ per 5 kg/m^2^ 0.73, 95% CI 0.52–1.03; OR_40_ per 5 kg/m^2^ 0.80, 95% CI 0.54–1.19), which did though not reach statistical significance. In comparison, with the exception of a BMI of ≥ 25 kg/m^2^ at baseline (HR 0.66, 95% CI 0.43–1.00), there was no significant association between BMI at age 40 or age 50 and clinical AD.

### Association of other risk factors with Aβ misfolding and clinical AD

The association of the remaining modifiable risk factors with Aβ misfolding and clinical AD is presented in Table 3. There was no significant association between diabetes, physical activity, smoking, and frailty index with Aβ misfolding. On the other hand, physical activity and frailty status were significantly associated with clinical AD incidence. More precisely, participants with low physical activity had a reduced risk of developing clinical AD within 17 years of follow-up by 43% (HR 0.57, 95% CI 0.39–0.85) compared to participants being physically inactive. Medium to high physical activity was nearly significantly associated with a reduced risk of clinical AD (HR 0.64, 95% CI 0.41–1.01). Being frail at baseline was associated with a more than doubled risk of developing AD (HR 2.02, 1.10–3.95), while no significant increase in the risk of clinical AD (HR 1.11, 0.71–1.72) was observed for being pre-frail compared to not being frail.

**Table 3:** Distribution of participant characteristics and their association with Aβ misfolding and clinical Alzheimer’s disease

		Aβ misfolding				clinical Alzheimer’s disease			
Characteristic at baseline		*N*_total_ (col %)	*N*_Abeta+_ (row %)	OR (95% CI)	*p*-value^b^	*N*_total_ (col %)	*N*_AD_ (row %)	OR (95% CI)	*p*-value^b^
				Adjusted^a^				Adjusted^c^	
Diabetes	No	686 (78.7)	100 (14.6)	Ref.		5066 (85.6)	116 (2.3)	Ref.	
	Yes	186 (21.3)	31 (16.7)	1.02 (0.59–1.77)	.9357	855 (14.4)	30 (3.5)	1.10 (0.65–1.87)	.7144
Physical activity	Inactive	278 (31.9)	35 (12.6)	Ref.		1110 (18.6)	49 (4.4)	Ref.	
	Low	420 (48.2)	68 (16.2)	1.44 (0.82–2.53)	.2088	2705 (45.3)	57 (2.1)	**0.57 (0.39–0.85)**	**.0056**
	Medium/high	173 (19.9)	28 (16.2)	1.14 (0.58–2.25)	.6978	2158 (36.1)	40 (1.9)	0.64 (0.41–1.01)	.0548
Smoking	Never	462 (54.9)	66 (14.3)	Ref.		2974 (50.8)	83 (2.8)	Ref.	
	Former	281 (33.4)	47 (16.7)	1.07 (0.61–1.87)	.8103	1975 (33.7)	42 (2.1)	0.89 (0.59–1.35)	.5957
	Current	98 (11.7)	17 (17.4)	1.19 (0.57–2.47)	.6452	908 (15.5)	16 (1.8)	1.07 (0.61–1.86)	.8212
Frailty index	Non-frail 0 to ≤0.20	206 (38.8)	31 (15.1)	Ref.		2337 (53.0)	39 (1.7)	Ref.	
	Pre-frail > 0.20 to < 0.45	253 (47.7)	50 (19.8)	1.14 (0.68–1.91)	.6132	1742 (39.5)	45 (2.6)	1.11 (0.71–1.72)	.6468
	Frail ≥0.45	72 (13.6)	9 (12.5)	0.75 (0.35–1.58)	.6452	330 (7.5)	15 (4.6)	**2.02 (1.10**–**3.95)**	**.0275**

^a^Adjusted for all variables listed in Table 1 and case/control status

^b^*p*-value derived from multiple logistic regression for adjusted odds ratios

^c^Adjusted for all variables listed in Table 1

## Discussion

This study focusing on the association between risk factors for Aβ misfolding in blood and AD found the presence of increased Aβ misfolding to be inversely associated with lower BMI at both baseline (mean age 68 years) and at age 50. The remaining risk factors for clinical AD were not significantly associated with Aβ misfolding.

### Association of risk factors with Aβ misfolding

The inverse association of the presence of increased Aβ misfolding with lower BMI even as early as at age 50 is a novel and potentially clinically highly relevant finding of our study. Higher late-life BMI has been reported to be associated with lower Aβ pathology by various studies [11, 20], and there have been suggestions that this association might reflect reverse causation rather than BMI effects [10, 21]. It has been concluded that weight loss might be an intrinsic pathological feature of Aβ accumulation where dementia-associated weight loss begins prior to clinical symptoms [22–24]. Furthermore, weight loss has been associated with AD CSF and imaging biomarkers in healthy elderly subjects [25]. Lower BMI in late life has been associated with greater cortical amyloid burden among clinically normal elderly [26]. On the other hand, higher midlife BMI has been shown to be associated with a higher risk of dementia [27] and cerebral amyloid deposition, although the association was not statistically significant in cognitively normal participants [28]. Furthermore, studies showed an association of midlife obesity with AD-pattern neurodegeneration but not amyloid deposition [29]; with earlier onset of AD; and with greater neuropathologic burden, including Braak neurofibrillary tangles [30]. Against this background, one could speculate that midlife obesity might be involved in the development of AD by pathways different from amyloid misfolding, such as vascular effects in the onset of AD and development of symptoms. All things considered, this result might reflect an association of unintentional weight loss (or reduced weight gain) with increased Aβ misfolding manifesting already in middle adulthood. This may be a very early sign of beginning AD pathology, particularly Aβ misfolding in blood. It cannot be ruled out that participants already showed unintentional weight loss as a symptom of early dementia but they had not yet received a diagnosis of dementia, which might not occur until a later stage when patients are unable to perform daily functions. It has also to be noted that this is the first study examining the relationship between BMI and Aβ misfolding. The longitudinal relationship between BMI and Aβ misfolding needs further evaluation in an ideally larger sample. In addition, collecting further blood biomarkers such as inflammatory cytokines and markers of oxidative stress could contribute to entangling this relationship as these have been linked to metabolic risk factors, including significant interactions towards cognitive decline [31, 32].

With regard to other common risk factors for clinical AD such as diabetes, physical activity, smoking, and frailty, we did not observe a significant association with increased Aβ misfolding. However, the longitudinal relationship of said factors with Aβ misfolding remains to be investigated.

### Association of risk factors with clinical AD

In the secondary analysis, the aforementioned risk factors and their association with clinical AD incidence were assessed. The main finding of this analysis was that low physical activity was associated with a reduced risk of developing clinical AD. The association of medium/high physical activity with reduced risk of developing clinical AD barely missed statistical significance, which was likely a result of the lack of power in that group. This is in line with previous research suggesting a benefit of physical activity on cognitive function and reduced incidence of AD [33, 34]. In consideration of the finding that physical activity was not associated with Aβ misfolding, this might elude to a role of physical activity in reducing the onset of clinical symptoms rather than the prevention of Alzheimer’s pathology. This is supported by a meta-analysis which found that even the cognitive function of AD patients can be improved by physical activity [35]. Additionally, participants who were classified as frail at baseline had a significantly higher risk of developing clinical AD. The association of frailty with clinical AD and the close connection between the two, including shared risk factors, has been well documented [36].

### Strengths and limitations

Limitations of this study include the limited power to detect weak-to-moderate associations, despite the overall large size of the cohort. In addition, the cross-sectional approach taken in the main part of the analysis precludes any conclusion about temporality or causality of associations. Regarding BMI, we relied on self-reported height and weight for all time points, which could have resulted in some misclassification. Due to the nature of a community-based cohort study, which portrays common practice rather than clinical evaluation in a highly specialized academic setting, misdiagnosis/underdiagnosis of clinical AD cannot be ruled out.

Strengths of this study include the population-based design, with data reflecting common general care practice in community settings, the use of an innovative test for measuring Aβ misfolding in blood, and the first-time assessment of the relationships between BMI and further modifiable risk factors of AD and Aβ misfolding in blood many years prior to clinical manifestation of AD.

## Conclusion

This study found Aβ misfolding, a very strong early marker of AD risk, not to be significantly associated with many risk factors of clinical AD. An exception was BMI, which was inversely associated with Aβ misfolding even as early as at age 50, a pattern that is consistent with AD-related weight loss (or reduced weight gain) that may be present many years before AD manifestation. Future studies with larger sample sizes should investigate the longitudinal relationship between modifiable risk factors and this early risk marker of AD for intervention and prevention measures.

## Supplementary Information

**Additional file 1: Table S1.** BMI measurements at different time-points according to Aβ misfolding and clinical Alzheimer’s disease status.

## Data Availability

The datasets used and/or analyzed during the current study are available from the corresponding author on reasonable request.

## Abbreviations

AD: Alzheimer’s disease
Aβ: Amyloid beta
BMI: Body mass index
CI: Confidence interval
CSF: Cerebrospinal fluid
ESTHER: Epidemiologische Studie zu Chancen der Verhütung Früherkennung und optimierten Therapie chronischer Erkrankungen in der älteren Bevölkerung
FI: Frailty index
GP: General practitioner
HR: Hazard ratio
iRS: Immuno-infrared-sensor
OR: Odds ratio
