# Supplementary material for: Aβ misfolding in blood plasma is inversely associated with body mass index even in middle adulthood
Source: Alzheimers Res Ther. 2021 Aug 30;13:145. doi: 10.1186/s13195-021-00889-2 (PMC8406782; doi:10.1186/s13195-021-00889-2)
Supplement: Supplementary file 1 — Additional file 1: Table S1. BMI measurements at different time-points according to Aβ misfolding and clinical Alzheimer’s disease status. [file 13195_2021_889_MOESM1_ESM.docx]

**Table S1.** BMI measurements at different time-points according to Aβ misfolding and clinical Alzheimer’s disease status.

| Time-point | Abeta misfolding | | |  | No abeta misfolding | | | p-value^a^ |  | Clinical Alzheimer’s disease | | | Dementia free | | | p-value^a^ |
| --- | --- | --- | --- | --- | --- | --- | --- | --- | --- | --- | --- | --- | --- | --- | --- | --- |
|  | N | Mean | SD |  | N | Mean | SD |  |  | N | Mean | SD | N | Mean | SD |  |
| Baseline | 127 | 27.1 | 3.8 |  | 719 | 28.0 | 4.2 | **.0276** |  | 143 | 27.2 | 3.9 | 5,739 | 27.7 | 4.5 | .1929 |
|  |  |  |  |  |  |  |  |  |  |  |  |  |  |  |  |  |
| At age 50 | 120 | 25.1 | 2.5 |  | 663 | 26.0 | 3.5 | **.0004** |  | 135 | 25.8 | 3.4 | 5,533 | 26.3 | 4.1 | .0951 |
|  |  |  |  |  |  |  |  |  |  |  |  |  |  |  |  |  |
| At age 40 | 112 | 24.0 | 2.3 |  | 644 | 25.6 | 3.2 | **.0208** |  | 125 | 24.4 | 3.1 | 5,484 | 24.8 | 3.6 | .1627 |

^a^ p-values derived from t-tests for continuous variables.

SD: Standard deviation.
